# The Future of Obesity Management through Precision Nutrition: Putting the Individual at the Center

**DOI:** 10.1007/s13668-024-00550-y

**Published:** 2024-05-28

**Authors:** Hande Gül Ulusoy-Gezer, Neslişah Rakıcıoğlu

**Affiliations:** Department of Nutrition and Dietetics, Faculty of Health Sciences, Hacettepe University, 06100 Sıhhiye, Ankara, Türkiye

**Keywords:** Obesity, Precision nutrition, Personalized nutrition, Polymorphisms, Weight loss, Dietary assessment

## Abstract

**Purpose of Review:**

The prevalence of obesity continues to rise steadily. While obesity management typically relies on dietary and lifestyle modifications, individual responses to these interventions vary widely. Clinical guidelines for overweight and obesity stress the importance of personalized approaches to care. This review aims to underscore the role of precision nutrition in delivering tailored interventions for obesity management.

**Recent Findings:**

Recent technological strides have expanded our ability to detect obesity-related genetic polymorphisms, with machine learning algorithms proving pivotal in analyzing intricate genomic data. Machine learning algorithms can also predict postprandial glucose, triglyceride, and insulin levels, facilitating customized dietary interventions and ultimately leading to successful weight loss. Additionally, given that adherence to dietary recommendations is one of the key predictors of weight loss success, employing more objective methods for dietary assessment and monitoring can enhance sustained long-term compliance.

**Summary:**

Biomarkers of food intake hold promise for a more objective dietary assessment. Acknowledging the multifaceted nature of obesity, precision nutrition stands poised to transform obesity management by tailoring dietary interventions to individuals' genetic backgrounds, gut microbiota, metabolic profiles, and behavioral patterns. However, there is insufficient evidence demonstrating the superiority of precision nutrition over traditional dietary recommendations. The integration of precision nutrition into routine clinical practice requires further validation through randomized controlled trials and the accumulation of a larger body of evidence to strengthen its foundation.

## Introduction

Obesity is a global epidemic that puts a tremendous burden on public health on account of its association with the increased risk of non-communicable diseases (NCDs). The European region, which consists of 53 countries in Europe and Central Asia, has high rates of overweight and obesity that affect almost 60% of adults and 30% of children, according to the WHO European Regional Obesity Report 2022. Unfortunately, the WHO Global Action Plan for preventing the rise in obesity prevalence has not been achieved by any European nation. The prevalence of obesity continues to increase exponentially regardless of age, sex, ethnicity, or socioeconomic status, and it shows no significant signs of decreasing soon [1•].

Obesity management strategies include a calorie-restricted diet, physical activity, behavior therapy, pharmacotherapy, and bariatric surgery. Clinical practice guidelines for overweight and obesity management highlight the importance of customized interventions and patient-centered care [2••]. Precision nutrition aims to personalize dietary recommendations based not only on phenotype but also on genotype or additional molecular factors such as gene expression, microbiome, proteome, and metabolome [3–5]. This novel approach can be considered to occur at three levels; Level 1 is the traditional diet based on general guidelines for populations by age, sex, and social determinants, Level 2 adds in phenotypic information about the individual’s current nutritional status (e.g. anthropometry, biochemical and metabolic analysis, physical activity), and Level 3 is genotype-oriented nutrition based on common or rare gene variations [6•].

All this information raises the question of how precision nutrition will affect obesity management. By synthesizing the existing literature, this review aims to contribute to the understanding of precision nutrition’s transformative potential in shaping the future of obesity management, with a focus on prioritizing personalized approaches.

## Methods of Literature Search

For this narrative review, we searched the PubMed, Science Direct, and Web of Science scientific databases and the gray literature including Google Scholar for English-language articles published between 2000–2023, with full-text availability. Data extraction was independently conducted by two researchers (HGUG and NR). The search approach involved of one or a combination of the following search terms, using the “AND” and “OR” operators: “precision nutrition”[TIAB], “personalized nutrition”[TIAB], “personalised nutrition”[TIAB], “obesity”[MeSH], “overweight”[MeSH], “weight loss”[MeSH], “body weight maintenance”[MeSH], “weight management”[TIAB], “polymorphism”[MeSH], “single nucleotide polymorphism”[MeSH], “gene variation”[TIAB], “biomarkers of food intake”[TIAB], “food intake biomarkers”[TIAB], “multiomics”[MeSH], “dietary assessment”[TIAB], “dietary monitoring”[TIAB], “nutritional status”[MeSH], “interindividual differences”[TIAB], “metabotype”[TIAB], “epigenomics”[MeSH], “genomics”[MeSH], “metabolomics”[MeSH], and “microbiomics”[TIAB]. To broaden the search, keywords with the asterisk (*) operator were employed. Additionally, hand-searching of reference lists from retrieved articles was also conducted. The process of selecting articles for inclusion involved thorough examination and discussion among the researchers to ensure relevance to the review's focus. Any discrepancies in article selection were resolved through consensus. After removing duplicates, a total of 85 peer-reviewed articles (excluding methodologic literature) were identified. These articles were then screened, and 13 were selected for detailed review and discussion, focusing on their contributions to understanding the connections between obesity and precision nutrition.

## Overview of Precision Nutrition

The aphorism "What is food to one, is to others bitter poison" uttered by Lucretius (99-55 BC), a Roman philosopher, was one of the first written sources to imply interindividual variability in response to dietary factors and their effects on human health [7]. Later, Roger J. Williams (1893–1988), who discovered pantothenic acid, noticed the differences among healthy individuals long before the relationship between nutrition and metabolism was established. While Williams' work provided early clues for precision medicine, it also demonstrated the need to address interindividual variability in response to nutrients, drugs, and vaccines [8]. Studies on the hypothesis that genetic differences contribute to interindividual nutrient, food, diet, and lifestyle responses have led to the emergence of precision nutrition concepts.

Current views on precision nutrition are based on omics technology, which aims to reveal all biological molecules involved in the structure, function, and dynamics of a cell, organism, or all organisms in a particular environment [6•]. These include a comprehensive analysis of genes (genomics), DNA modifications (epigenomics), messenger RNA (mRNA) or transcripts (transcriptomics), proteins (proteomics), metabolites (metabolomics), lipids (lipidomics), foods (foodomics), and microbiota (microbiomics, metagenomics) [9]. Significant connections exist between bioactive food components and cellular processes, with changes apparent at different molecular levels, encompassing DNA (nutrigenetics), pre-transcriptional modifications (such as methylation and epigenetics) affecting mRNA (nutrigenomics), and proteins (proteomics) [10]. Since single-omics technology alone cannot represent the whole picture of nutrition and obesity, the use of “multi-omics” technology has been accepted to provide a more comprehensive view [9]. Recent advances in multi-omics technology have shed light on the molecular and cellular effects of nutrients on individuals, enabling personalized nutritional interventions that not only consider physiological, ethnic, cultural, and economic factors but also take into account genomics, foodomics and microbiomics [10].

**Fig. 1:**
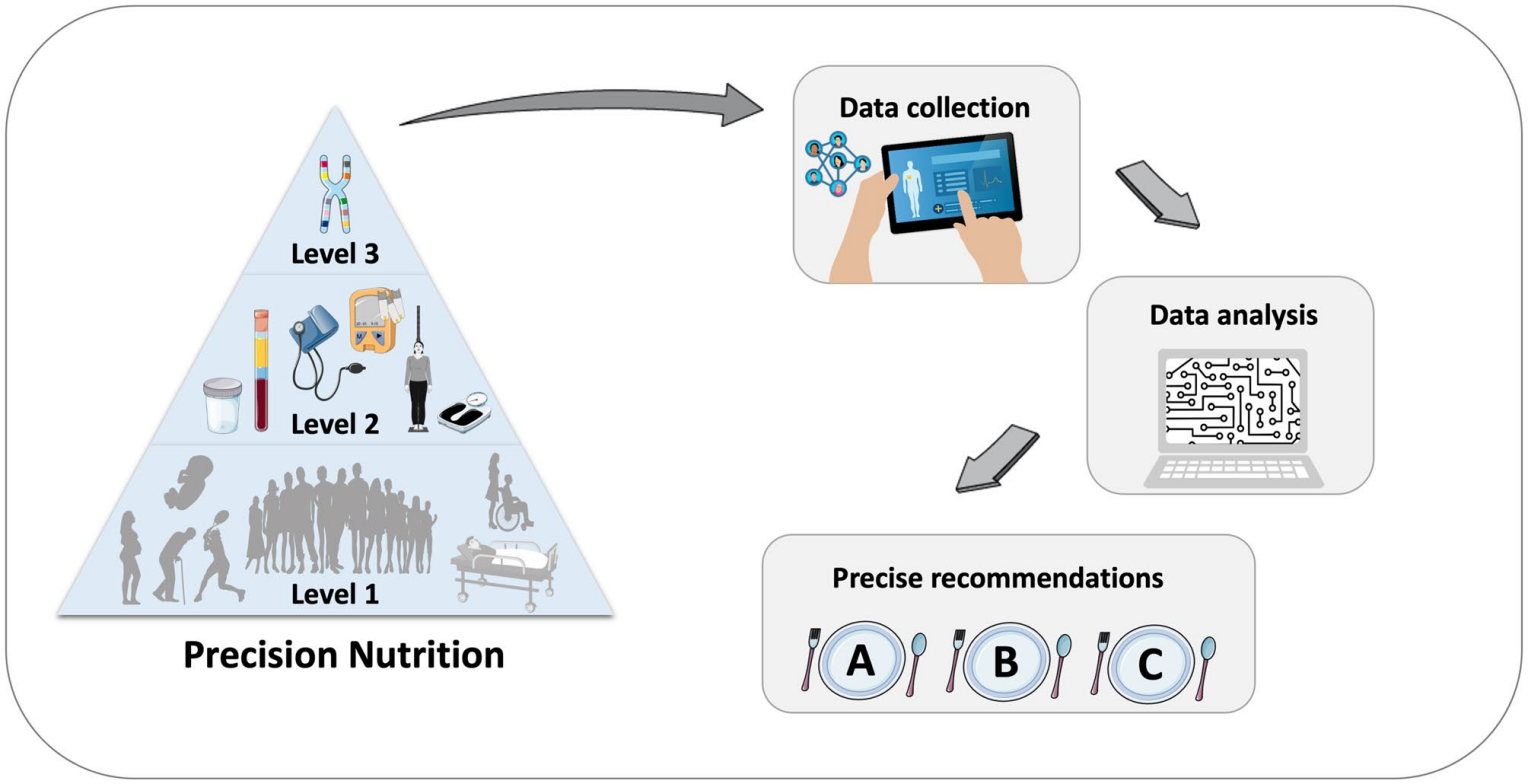
Precision nutrition and “metabotyping”. The figure was modified with text and after adaptation of images from Servier Medical Art by Servier, licensed under a Creative Commons Attribution 3.0 Unported License (https://smart.servier.com)

Nevertheless, using these technologies for each individual is expensive and time-consuming. Metabotyping, clustering individuals into three subgroups based on their similar metabolic phenotypes (anthropometric, biochemical, metabolomics, and microbiomics data), is a more feasible and cost-effective option for precision nutrition strategy at the group level (Fig. 1) [11–14].

## Genetics of Obesity

The genome is defined by all the genetic material, which consists of 23 pairs of chromosomes in the nucleus and a small circular chromosome in the mitochondria called mitochondrial DNA (mtDNA). Despite phenotypic diversity, the human genomes are substantially 99.9% identical, implying that only minor differences in DNA sequence exist [15]. Understanding the human genome has been a journey toward gaining a better understanding of interindividual differences. The success of the Human Genome Project, an international research project aimed at identifying the entire human DNA sequence through the sequencing of 3 billion base pairs, led to more rapid and less expensive DNA sequencing techniques.

The minor differences in DNA are commonly referred to as mutations or polymorphisms. Mutations, which are permanent alterations in the DNA sequence, occur at < 1% frequency in the population and most certainly cause disease by impacting phenotype [16]. Polymorphism refers to the common variant in a specific DNA sequence or the presence of two or more alleles of the gene in a locus in the population at a frequency of approximately ≥ 1%. Polymorphism does not directly cause disease but may act as a predisposing factor [17]. Single nucleotide polymorphism (SNP), the most common type of polymorphism, involves a change in a single base pair. Although the human genome normally contains approximately 3–10 million SNPs, most SNPs do not affect phenotype [18]. Some SNPs, which are in coding regions of DNA, can affect protein synthesis and function, altering nutrient requirements and metabolism [6•].

In obesogenic settings, 40% to 70% of the interindividual differences are attributed to genetic factors [19]. From the framework of genetics, obesity can be described as two categories, monogenic and polygenic obesity. Monogenic obesity is characterized as severe obesity with early-onset caused by a single gene mutation/deficiency. For example, mutations in the genes encoding melanocortin 4 receptor (*MC4R*), leptin receptor (*LEPR*), and pro-opiomelanocortin (*POMC*), result in monogenic obesity. While monogenic obesity is rare, polygenic obesity, caused by hundreds of variants in many genes, is common among individuals with obesity. Each gene variant has a minor impact, as opposed to monogenic obesity the environment largely determines the development of polygenic obesity [20••, 21••].

Over the last decade, the field of multifactorial disease genetics has been changed by genome-wide association studies (GWAS) which aim to determine genotype–phenotype relationships related to disease susceptibility by identifying genomic variants. Although more ethnically diverse studies with larger sample sizes are still lacking, GWAS help identify novel variant-disease or trait associations or novel mechanisms behind them [22]. GWAS have contributed to the identification of obesity-associated genes such as *FTO* (Fat mass and obesity-associated protein) [23, 24, 25•, 26, 27], *MC4R* (Melanocortin 4 receptor) [28], *MTHFR* (Methylenetetrahydrofolate reductase) [29], *LEP* (Leptin), *LEPR* (Leptin receptor), *ADIPOQ* (Adipocyte C1q and collagen domain containing), *IL* (Interleukin), *TNF-α* (Tumour necrosis factor-α) [30], *BDNF* (Brain-derived neurotrophic factor) [31], *ADRB2* (Beta2-adrenergic receptor) [32], *ADCY3* (Adenylate cyclase) [27], *PPAR-γ* (Peroxisome proliferator-activated receptor-gamma) [33], *PCSK1* (Proprotein convertase subtilisine/kexin type 1) [34], *VDR* (Vitamin D receptor) [35], *TMEM18* (Transmembrane protein 18) [36], and *CLOCK* (Circadian locomotor output cycles kaput) [37, 38•]. Among the hundreds of obesity-associated genes, *FTO* has been identified as the most robust predictor of polygenic obesity [21••].

These obesity-associated genes have been very important in elucidating physiological mechanisms. However, the same polymorphisms in the same gene can even show differences between the genders in the same population. Such as risk variants in *FTO*, a gene known to be involved in appetite control and eating behavior, is linked to a higher risk of obesity due to increased food intake and greater preference for foods with high energy and fat in children and adults but not in older adults [39, 40•]. Similarly, the *LIPC* (Hepatic lipase) gene C-514 T polymorphism among Chinese children was associated with higher body mass index (BMI) levels in boys, and it was found to protect against higher BMI levels in girls [41].

With the increasing number of studies and advanced technological developments, the identification of obesity-associated candidate genes is growing rapidly. Since genomic data is large and complex, analytical tools such as machine learning algorithms and deep learning methods are required to reveal unexpected genomic relationships, derive hypotheses and new models, and make predictions. Machine learning algorithms are suitable for data-driven sciences such as genomics, as they are designed to automatically detect patterns in data [42]. Associations of some SNPs with obesity from meta-analyses of case-control studies are shown in Table 1. Given the pivotal role of genetic information in precision nutrition at level 3, researchers have undertaken precision nutrition studies that integrate some of these specific genes and related SNPs to personalize interventions. This emphasis is exemplified by studies summarized in Table 3, where the utilization of SNPs emerges as a prominent strategy for personalized interventions. The likelihood that genetic data may soon be utilized to determine people with a higher risk of obesity is increasing as more variations for obesity are uncovered. Unfolding if there is a genetic vulnerability, would give the chance to act earlier to prevent obesity [21••].

**Table 1:** Associations of single nucleotide polymorphisms with obesity from meta-analyses of case-control studies

**Gene**	**SNP**	**Sample Size**		**Age Group**	**Ethnicity**	**Reference**
		**Cases**	**Controls**			
*ADIPOQ*	rs2241766	2819	3024	Adults	Chinese	Wu et al. [43]
*ADRB3*	rs4994	5147	7350	Children, adolescents	East Asians	Xie et al. [44]
*ENPP1/PC-1*	rs1044498	11372	12952	Adults	Europeans	Wang et al. [45]
*FABP2*	rs1799883	569	446	Adults	Asians	Shabana and Hasnain [46]
*FTO*	rs1421085	5169	7772	Adults	Caucasians, Asians,	Najd-Hassan-Bonab et al. [25•]
*FTO*	rs9939609	5000	9853	Children, adolescents	Caucasians, Amerindians, Asians	Quan et al. [26]
*FTO*	rs9939609	7311	8302	Children, adolescents	Chinese, Europeans, Indians, Chileans	da Silva et al. [23]
*FTO*	rs9930506	3337	3159	Adults, children	Europeans	Doaei et al. [24]
*IL-6*	rs1800795	4291	2919	Adults, adolescents	Caucasians	Hu et al. [47]
*MC4R*	rs2229616	19822	35373	Adults, children	East Asian	Wang et al. [48]
*MC4R*	rs17782313	80957	220223	Adults, children	Europeans, East Asians	Xi et al. [49]
*MC4R*	rs17782313	3133	3123	Adults	Europeans, Asians	Yu et al. [28]
*MTHFR*	rs1801133	8622	29695	Adults, children	Asians, Europeans, Chinese, African, Mestizo	Fu et al. [29]
*PPAR-γ2*	rs1801282	6491	8242	Adults	Caucasians, Asians, Mixed	Yao et al. [33]
*RETN*	rs1862513	5069	6673	Adults	Chinese, Europeans, Arabians	Zhu et al. [50]
*UCP2*	rs659366	8652	10075	Adults, adolescents, children	Asians, Africans	Abd El Daim et al. [51]
*UCP2*	rs659366	7647	11322	Adults, children	Europeans	Liu et al. [52]
*UCP2*	rs659366	7390	9860	Adults	Europeans	Qian et al. [53]

*ADIPOQ* Adiponectin, *ADRB3* Adrenoceptor beta 3, *ENPP1/PC-1* Ectonucleotide pyrophosphatase/phosphodiesterase 1/Plasma-cell membrane glycoprotein-1, *FABP2* Fatty acid binding protein 2, *FTO* Fat mass and obesity-associated protein, *IL-6* Interleukin-6, *MC4R* Melanocortin-4-receptor, *MTHFR* Methylenetetrahydrofolate reductase, *PPAR-γ2* Peroxisome proliferator-activated receptor-γ2, *RETN* Resistin, *SNP* Single nucleotide polymorphism, *UCP2* Uncoupling protein 2

## Precision Nutrition for Obesity

**Fig. 2:**
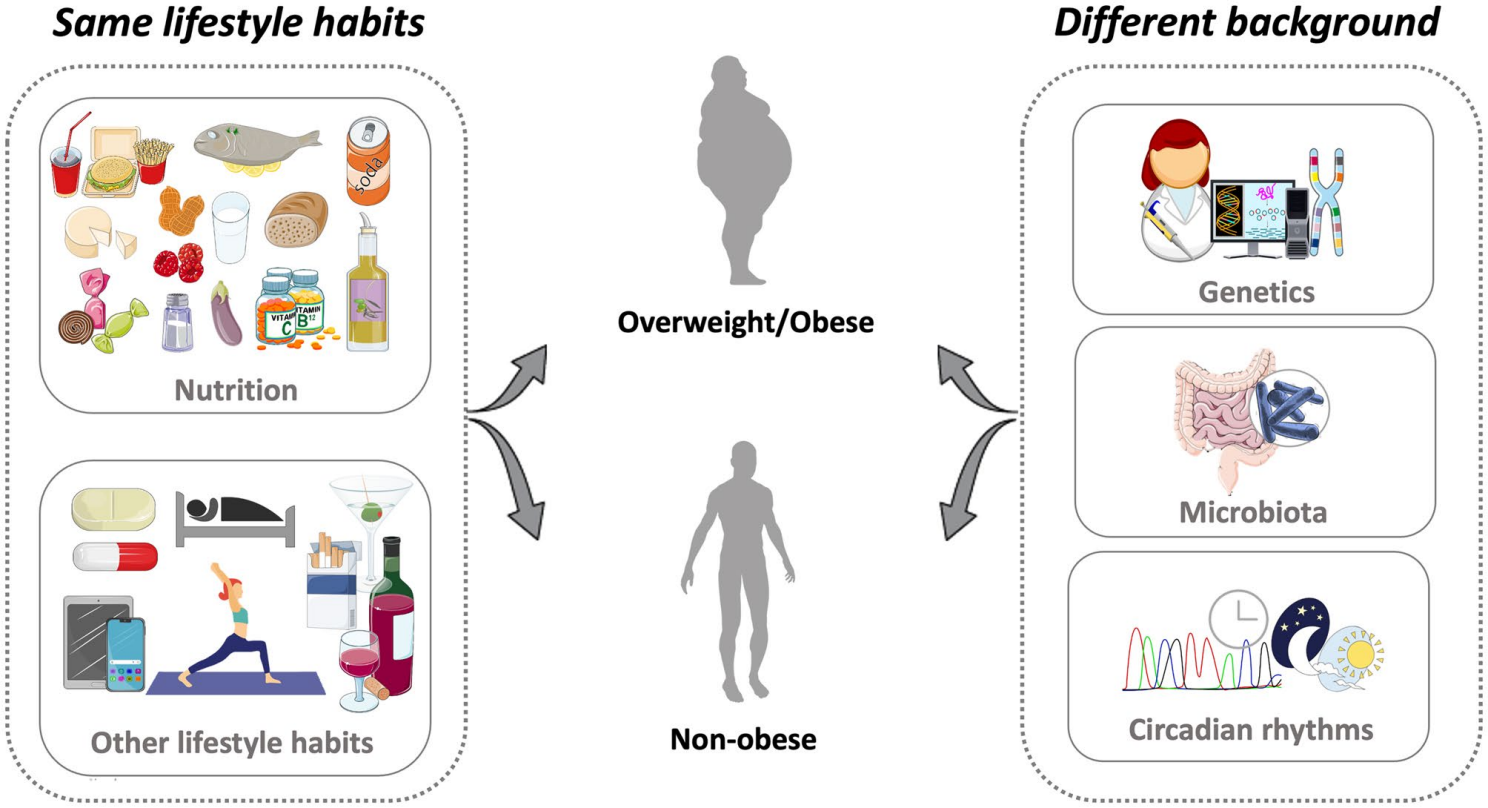
Interindividual variability in response to same dietary pattern, and lifestyle habits. The figure was modified with text and after adaptation of images from Servier Medical Art by Servier, licensed under a Creative Commons Attribution 3.0 Unported License (https://smart.servier.com)

Low-calorie diets with different macronutrient compositions, such as low-fat, low-carbohydrate, or high protein, has been investigated for years to find optimal diet strategy for weight loss. Across all dietary interventions, significant variability in weight loss is observed [54]. Lately, obesity studies’ focus has shifted from modeling the metabolic effects of macro- and micronutrients to meal frequency and timing (intermittent fasting), eating window (early or late time-restricted eating), sleep quantity and quality, postprandial responses through circadian rhythms, and genetic variants to overcome the interindividual differences in weight loss [55, 56••, 57•, 58]. If more objective and precise data is collected from individuals, the basis of interindividual differences can be understood, and thereby tailored recommendations can be offered for weight loss optimization, weight regain prevention, and long-term weight loss maintenance (Fig. 2). However, it's essential to ensure that these recommendations align with the individual food preferences, economic and social circumstances, food accessibility, culinary expertise, and other relevant considerations [54, 55, 56••].

## Personalized Data Collection and Analysis

Precision nutrition requires an in-depth quantitative level of information from genetics and digital health profiles using technology-based assessment methods for dietary intake, physical activity (e.g. Fitbit, Garmin, Polar), flash (e.g. Abbott FreeStyle Libre), or real-time blood glucose levels (e.g. Nintamed Dexon G6, Medtronic Guardian Connect, Senseonics Eversense), blood pressure (e.g. Qardio Arm, Omron RS8, HeartGuide, CNAP2GO), heart health (e.g. Qardio Chest, iHeart) and sleep (e.g. ActiGraph, Fitbit, Garmin, Fatigue Science) [59, 60, 61•, 62–64]. Many physiological markers can now be monitored for patients outside of clinics thanks to advancements in wearable and non-invasive sensors. The Novel Coronavirus Disease (COVID-19) epidemic's rules requiring social distance and home isolation have once again highlighted the significance of telehealth programs that provide home screening, diagnosis, and monitoring. Improving patient monitoring with these methods can help alleviate the overcrowding of hospitals and the increasing workload of healthcare professionals, which are among the biggest issues of the healthcare system.

## Dietary Assessment

Dietary history, food record, 24-h dietary recall, and food frequency questionnaire (FFQ), subjective dietary assessment methods, are widely used in human nutritional studies [65]. However, these methods are all to some extent subjective and in general are biased due to misreporting or underreporting of dietary intake and is affected by individual characteristics such as age, gender, and BMI [66, 67]. While adults with overweight or obesity tend to under-report, adults with underweight tend to over-report [66]. Additionally, the reliability of information obtained from the elderly may decrease with increasing age of the respondent due to cognitive impairments (memory loss, decreased cognitive function, common neurological diseases such as dementia), reductions in functional capacity (problems with vision, hearing, and writing), reduced or absent participation in food-related processes [67]. Also, accurate dietary assessment is challenging in children due to the lack or limited literacy and writing skills, limited food recognition and portion size estimation skills, memory limitations, and short focusing time [68]. Therefore, both in clinical and non-clinical settings, more objective dietary measurements are needed for the implementation of precision nutrition across all age groups [69]. Web-based, image-assisted, and image-based dietary assessment methods may be more useful than traditional dietary assessment methods for patient monitoring outside clinical settings, as they offer remote access. Furthermore, image-assisted and image-based methods may prevent misreporting [70].

### Web-based Dietary Assessment

The increase in internet use in recent years has popularized web-based dietary methods for both research and individual purposes. While most of the web-based dietary assessment tools developed are based on 24-h dietary recall and FFQ, some are based on food records or diaries. These methods can be supplemented with images to improve the estimation of portion size. They provide a cost-effective collection of data in large-sample studies [71]. It has been shown that web-based tools such as FIRRSt (Food Intake Recording Software System), and INTAKE24, for measuring dietary intake in children are often more engaging than traditional, paper-based methods and keep the child focused. Therefore, it may be beneficial to prefer web-based methods for assessing nutritional status, especially in children [72]. Although childhood and adolescence are important periods for the development of obesity, few studies have focused on these periods [73].

### Image-assisted Dietary Assessment

Image-assisted dietary assessment refers to any method that uses images or videos of mealtimes to improve the reliability of traditional methods. It is a new technology that uses food recognition, segmentation, classification, volume estimation, and deep learning techniques via mobile phones, depth cameras, RGB (red-green-blue) cameras, wearable sensors, or smart glasses [74]. Images can be captured in two different approaches, active and passive [70]. The active approach requires individuals to take images with a handheld device such as a digital camera or smartphone. Images are usually taken before and after meals to distinguish the leftovers, holding the device at an angle of about 45°. A reference marker of known dimensions and color is placed on each image. Images are often supplemented with additional text or audio recordings describing the foods [75•]. The passive approach does not depend on users capturing images of meals. It requires wearable cameras to automatically capture images of everyday events, including mealtimes. However, the captured images are not only for mealtimes, and this may not be accepted by everyone due to privacy concerns [70].

### Image-based Dietary Assessment

While image-assisted methods are used to assist traditional dietary assessment methods or to remember the foods consumed, image-based methods are the primary recording method of dietary assessment. Images can be actively captured by the individual using a camera or smartphone, or passively recorded by a wearable device, as in image-assisted methods [70].

## Dietary Monitoring Techniques

**Fig. 3:**
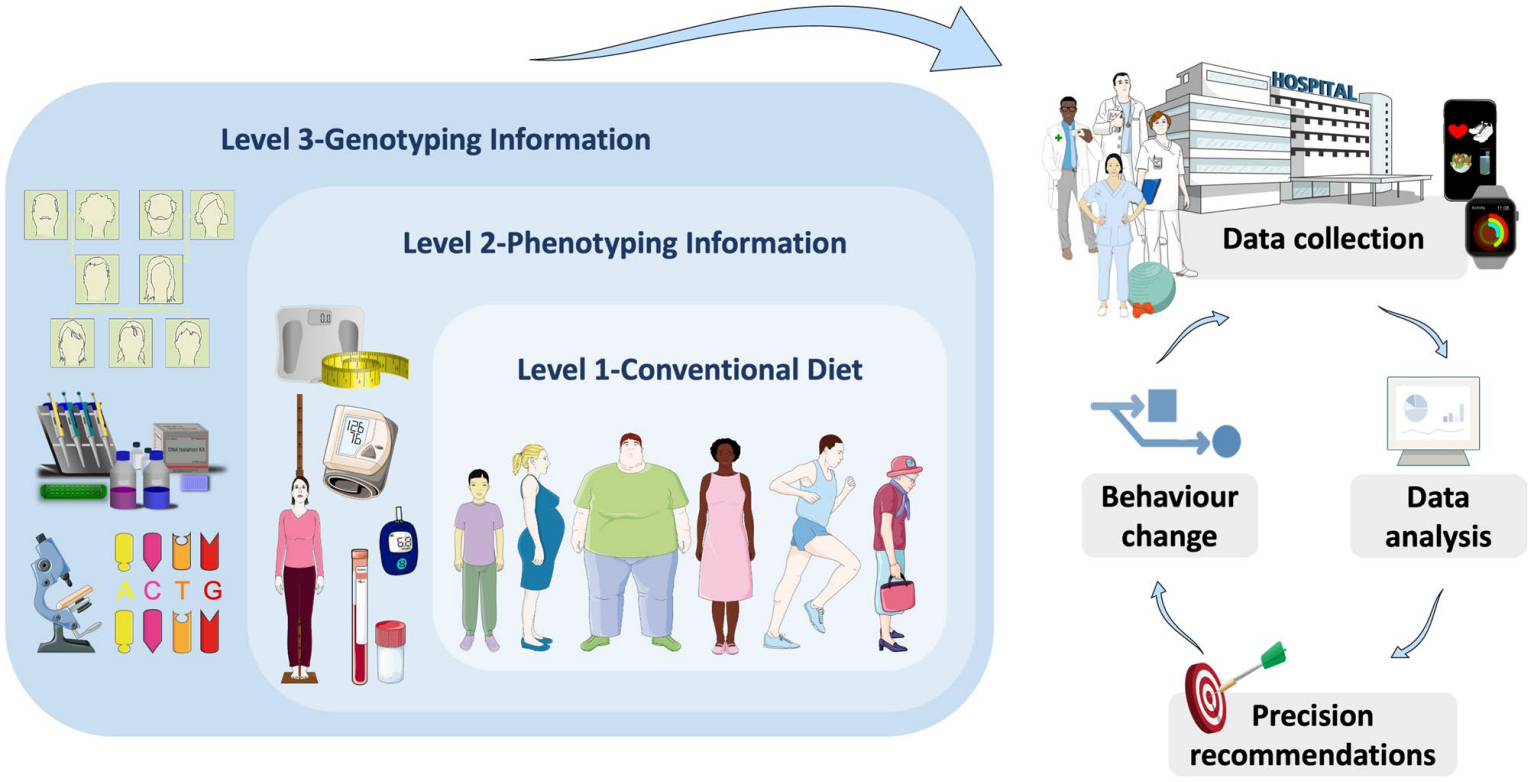
Precision nutrition can be considered to occur at three levels: (1) conventional diet based on individuals’ age, gender, and other determinants, (2) phenotypic information such as anthropometry, biochemical and metabolic markers, (3) genotypic information based on omics technologies*.* The figure was modified with text and after adaptation of images from Servier Medical Art by Servier, licensed under a Creative Commons Attribution 3.0 Unported License (https://smart.servier.com)

Motivating people to alter their lifestyle, including eating habits, is the largest barrier in improving any behavior. By continuously monitoring individuals outside of the clinics, these monitoring techniques can boost motivation and compliance (Fig. 3). There is a growing interest in smartphone-based self-monitoring apps such as My Meal Mate and My Fitness Pal. Although these apps sometimes lack evidence-based content, potentially limiting their efficacy, they can also provide rapid and personalized awareness through direct feedback [76]. The biggest advantage of monitoring via smartphone is that all the computing power required for data processing, hardware such as a camera, display for test results, a power supply, and most of the required auxiliary support is already available in an owned smartphone [77].

As dietary assessment methods evolve, so does the demand for comprehensive and precise techniques to monitor dietary intake. Although some are only in the project phase for now, smart plate [78], smart dining tray [79], wearable sensors such as wrist-worn devices (e. g. smartwatch-based eating detection) for monitoring energy intake [80], smart fork for monitoring eating rate [81], or smart water bottle for monitoring water intake are other novel monitoring techniques [82]. Moreover, with technological advances, ingestible sensors are being developed that can measure parameters such as pH, temperature, pressure, gas, microbial population, and digestion and fermentation metabolites [83].

## Biomarkers of Food Intakes

Metabolomics aims to determine the metabolome, considered a chemical representation of a biological phenotype. Blood (plasma or serum), saliva, urine, faces, tissue, exhaled breath, hair, and nail are frequently used biological samples in metabolomics studies [84, 85]. However, there are differences between using urine and blood (plasma or serum) as biological samples. Urine collection is less expensive and more practical than blood collection, enables quick collection of large quantities, and is less invasive for the subject. More than 4000 metabolites have been detected in the urine, although not all of them are related to dietary intake [86]. Urine is rich in non-nutrient bioactive compounds such as phytochemicals and their metabolites, and most of these metabolites are excreted rapidly. Blood, on the other hand, has a higher concentration of metabolically active chemicals, and fat-soluble metabolites are only found in plasma. Moreover, urinary biomarkers are considered short-term biomarkers of food intake (BFIs) [87]. Urinary naringenin and hesperetin are examples of short-term biomarkers for citrus fruits especially, orange juice intake [88]. As an exception, Saenger et al. showed that urinary proline betaine is traceable for at least 72 h after orange juice intake, and it is identified as a relatively long-term biomarker [89].

Table 2 summarizes some of the potential biomarkers of food intake from human studies. According to the scoring system developed by Rafiq et al. 69 metabolites were identified from 11 food-specific categories or dietary patterns that scored ≥ 5 points pointing to good evidence as a potential BFI. Through BFIs, information can be obtained not only on specific foods but also on dietary patterns. For example, it has been shown that 24-h urinary N-(4-methyl-5-oxo-1-imidazolin-2-yl)sarcosine (MG-HCr) excretion differs in individuals with different dietary habits such as omnivores (0.39 − 9.67 μmol/day), vegetarians (0.18 − 0.97 μmol/day), and vegans (0.10 − 0.27 μmol/day) [90]. Treibmann et al. suggested that urinary MG-HCr could be used as a short-term biomarker for heat-treated animal food intake [91].

**Table 2:** Potential biomarkers of food intake from human studies

**Food**	**Biomarker**	**Biological Sample**	**Sample Size**	**Food Survey**	**Analytical Procedure**	**Reference**
***Meats***						
Chicken	3-methylhistidine	Plasma	10 adults	24-h dietary recall	^1^H-NMR	Cheung et al. [92]
	3-methylhistidine	Plasma	10 adults	Dietary intervention	^1^H-NMR	Yin et al. [93]
	Dimethylglycine	Plasma	18 adults	Dietary intervention	LC–MS/MS	Giesbertz et al. [94]
	π-methylhistidine	Plasma	294 adults	24-h dietary recall for 3-d	LC–MS/MS	Mitry et al. [95]
	Anserine	24-h urine	10 adults	24-h dietary recall	^1^H-NMR	Cheung et al. [92]
	Guanidoacetate	24-h urine	10 adults	Dietary intervention	^1^H-NMR	Yin et al. [93]
Fish	Trimethylamine-N-oxide	Plasma	10 adults	24-h dietary recall	^1^H-NMR	Cheung et al. [92]
	Trimethylamine-N-oxide	24-h urine				
Herring, mackerel	1-methylhistidine	Plasma	232 children	Dietary intervention	GC–MS	Solvik et al. [96]
Meat	2-methylbutyrylcarnitine	Plasma	10 adults	24-h dietary recall	^1^H-NMR	Cheung et al. [92]
	Acetylcarnitine	Plasma				
	Anserine	Plasma	18 adults	Dietary intervention	LC–MS/MS	Giesbertz et al. [94]
	Carnosine	Plasma	294 adults	24-h dietary recall for 3-d	LC–MS/MS	Mitry et al. [95]
	Propionyl carnitine	Plasma	10 adults	24-h dietary recall	^1^H-NMR	Cheung et al. [92]
	Trimethylamine-N-oxide	Plasma	18 adults	Dietary intervention	LC–MS/MS	Giesbertz et al. [94]
	π-methylhistidine	Plasma	294 adults	24-h dietary recall for 3-d	LC–MS/MS	Mitry et al. [95]
	π-methylhistidine	Plasma	18 adults	Dietary intervention	LC–MS/MS	Giesbertz et al. [94]
	1-methylhistidine	24-h urine	17 adults	Dietary intervention	IEX chromatography	Cross et al. [97]
	3-methylhistidine	24-h urine				
	Carnosine	24-h urine	10 adults	24-h dietary recall	^1^H-NMR	Cheung et al. [92]
Poultry	pi-methylhistidine	Plasma	294 adults	24-h dietary recall for 3-d	LC–MS/MS	Mitry et al. [95]
Salmon	1-methylhistidine	Spot urine	24 adults	Dietary intervention	FT-ICR-MS	Lloyd et al. [98]
	Trimethylamine-N-oxide	Spot urine				
***Dairy and plant-based milk***						
Cheese	Heptan-2-one	Plasma	11 adults	Dietary intervention	GC–MS	Fuchsmann et al. [99]
	Undecan-2-one	Plasma				
	Heptan-4-one	24-h urine				
Dairy	3,5-dimethyloctan2-one	Plasma				
	1-methoxy-2-propyl acetate	24-h urine				
	3-ethylphenol	24-h urine				
	9-decenoic acid methyl ester	24-h urine				
Soy-based milk	1-octen-3-ol	24-h urine				
	1,3-octadiene	24-h urine				
	2,4-octadiene	24-h urine				
	Acetophenone	24-h urine				
	Methoxycyclooctane	24-h urine				
***Grains***						
Whole grain	Alkylresorcinol	Plasma	33 adults	3-d food diaries	LC–MS/MS	Ampatzoglou et al. [100]
Whole grain wheat, rye	3,5-dihydroxycinnamic acid	24-h urine	69 adults	3DWFR	LC–MS/MS	Wierzbicka et al. [101]
	5-(3,5-dihydroxyphenyl)pentanoic acid	24-h urine				
***Fruits and fruit juices***						
Apple, pear	Phloretin glucuronide	24-h urine	481 adults	24-h dietary recall, FFQ	UPLC-qTOF-MS	Edmands et al. [102]
Aronia-citrus juice	Ferulic acid	Spot urine	51 adults	Dietary intervention	UPLC-qTOF-MS	Llorach et al. [103]
	Proline betaine	Spot urine	51 adults	Dietary intervention	UPLC-qTOF-MS	Llorach et al. [103]
Citrus fruits	Hesperetin glucuronide	24-h urine	481 adults	24-h dietary recall, FFQ	UPLC-qTOF-MS	Edmands et al. [102]
	Naringenin glucuronide	24-h urine				
	Hesperetin glucuronide	24-h urine for 3-d	107 adults	3DWFR	UPLC-qTOF-MS	Andersen et al. [104]
	Proline betaine	24-h urine for 3-d				
Citrus fruits and juices	Flavanones	24-h urine	475 adults	24-h dietary recall, FFQ	HPLC–MS/MS	Tahiri et al. [105]
	Hesperetin	24-h urine				
	Naringenin	24-h urine				
Grape	Tartaric acid	24-h urine	19 adults	Dietary intervention	^1^H-NMR	Garcia-Perez et al. [106]
Raspberry	Sulphonated caffeic acid	Spot urine	24 adults	Dietary intervention	FT-ICR-MS	Lloyd et al. [98]
	Sulphonated methyl-epicatechin	Spot urine				
Strawberry	2,5-Dimethyl-4-methoxy-3(2H)-furanone sulphate	24-h urine for 3-d	107 adults	3DWFR	UPLC-qTOF-MS	Andersen et al. [104]
***Vegetables and vegetable juices***						
Beetroot	4-Ethyl-5-methylamino-pyrocatechol sulphate	24-h urine for 3-d	107 adults	3DWFR	UPLC-qTOF-MS	Andersen et al. [104]
	4-Methylpyridine-2-carboxylic acidglycine conjugate	24-h urine for 3-d				
Cruciferous vegetables	S-methyl-L-cysteine sulfoxide	48-h urine	20 adults	Dietary intervention	^1^H-NMR	Edmands et al. [107]
Dark green vegetables	α-Carotene, β-Carotene, β-Cryptoxanthin, Lutein, Zeaxanthin	Serum	147 adults	R24W for 4-d	HPLC	Lafrenière et al. [108]
Green beans	Unsaturated aliphatic hydroxy-dicarboxylic acid	24-h urine for 3-d	107 adults	3DWFR	UPLC-qTOF-MS	Andersen et al. [104]
Orange vegetables	α-Carotene, β-Carotene, β-Cryptoxanthin, Lutein, Zeaxanthin	Serum	147 adults	R24W for 4-d	HPLC	Lafrenière et al. [108]
Red bell pepper (fresh)	Capsanthin	Spot urine	14 adults	Dietary intervention	^1^H-NMR	Schulz et al. [109]
Red cabbage, Brussels sprouts, pointed cabbage	*N-*acetyl-S-(*N*-3-methylthiopropyl)cysteine	24-h urine for 3-d	107 adults	3DWFR	UPLC-qTOF-MS	Andersen et al. [104]
	Iberin *N*-acetyl-cysteine	24-h urine for 3-d	107 adults	3DWFR	UPLC-qTOF-MS	Andersen et al. [104]
Red cabbage, Brussels sprouts, horseradish	*N*-acetyl-S-(*N*-allylthiocarbamoyl)cysteine	24-h urine for 3-d				
Red cabbage	3-Hydroxy-3-(methyl-sulphinyl)propanoic acid	24-h urine for 3-d	107 adults	3DWFR	UPLC-qTOF-MS	Andersen et al. [104]
	3-Hydroxy-hippuric acid sulphate	24-h urine for 3-d				
Tomato juice	Steroidal alkaloids	48-h urine	14 adults	Dietary intervention	LC–MS/MS	Hövelmann et al. [110]
	β-carboline	48-h urine				
	Imidazole	48-h urine				
***Sugars***						
Chocolate	4-Hydroxy-(3’,4’-dihydroxyphenyl)valeric acid sulfate	24-h urine	481 adults	24-h dietary recall, FFQ	UPLC-qTOF-MS	Edmands et al. [102]
***Nuts***						
Walnut	5-Hydroxyindole-3-acetic acid	24-h urine for 3-d	107 adults	3DWFR	UPLC-qTOF-MS	Andersen et al. [104]
***Drinks***						
Coffee	AAMU	Serum	451 adults	24-h dietary recall, FFQ	MS/MS	Rothwell et al. [111]
	Caffeine	Serum				
	Cyclo(isoleucyl-prolyl)	Serum				
	Cyclo(prolyl-valyl)	Serum				
	Paraxanthine	Serum				
	Pyrocatechol sulfate	Serum				
	Quinic acid	Serum				
	Trigonelline	Serum				
	Dihydroferulic acid sulfate	24-h urine	481 adults	24-h dietary recall, FFQ	UPLC-qTOF-MS	Edmands et al. [102]
	Ferulic acid sulfate	24-h urine				
	Guaiacol glucuronide	24-h urine				
Red wine	Gallic acid ethyl ester sulfate	24-h urine				
Tea	Methylgallic acid sulfate	24-h urine				
***Others***						
Phytosterols	Campesterol:5-α-cholestanol ratio	Plasma	38 adults	Dietary intervention	GC–MS	Lin et al. [112]

^*1*^*H-NMR* hydrogen-1 nuclear magnetic resonance, *3DWFR* 3-d weighed dietary records, *AAMU* 5-acetylamino-6-amino-3-methyluracil, *FFQ* Food Frequency Questionnaire, *FT–ICR–MS* Fourier transform–ion cyclotron resonance ultra-mass spectroscopy, *GC–MS* gas chromatography—mass spectrometry, *HPLC–MS/MS* High-performance liquid chromatography–mass spectrometry, *IEX* Ion exchange, *LC–MS/MS* Liquid chromatography–mass spectrometry, *MS/MS* Tandem mass spectrometry, *R24W* Web-based 24-h dietary recall, *UPLC–qTOF–MS* ultra-high performance liquid chromatography-quadrupole time-of-flight mass spectrometry

Most food-specific metabolites are detected in human blood and urine for 5 to 10 h, with others detected for up to 48 h. It is recommended to use a model of at least 24–48 h in which several biological samples are obtained to assess variations in metabolite concentration over time or to produce an average value representative of the genuine concentration [90]. In general, to assess potential BFIs, two different metabolomic techniques are used: (a) mass spectrometry (MS) combined with gas (GC-MS, gas spectrum-MS) or liquid phase chromatography (UPLC-qTOF-MS, ultra-high-performance liquid chromatography-quadrupole time-of-flight-MS), and (b) proton nuclear magnetic resonance (^1^H-NMR) spectroscopy [88]. Although ^1^H-NMR-based techniques are non-destructive and offer greater reproducibility, and less inter-laboratory variance, these techniques have a lower sensitivity compared to MS-based approaches [113]. Thereby, NMR is best at detecting high amounts of metabolites, while GC–MS and UPLC-qTOF-MS are best at low amounts [114•]. Otherwise, the efficacy of BFIs may vary depending on the diurnal variance, the day of the week, or the season of sample collection. Sample collection procedures, transportation, processing, storage settings, and storage period of acquired biological samples are also effective aspects in determining the efficacy of possible BFIs [115].

Cheung et al. showed urinary and plasma trimethylamine N-oxide (TMAO) as a potential biomarker for fish intake in a controlled dietary intervention study [92]. Yin et al. reported that although urinary TMAO showed a strong dose-response relationship with fish consumption (r = 0.148, p < 0.01), the use of urinary TMAO alone to determine fish intake in a free-living population was insufficient [116]. Combined use of BFIs should also be considered, as the combinations of BFIs may be more effective in predicting food intake rather than using one biomarker alone [117]. Databases of dietary phytochemicals and their metabolites in humans such as Phenol-Explorer, PhytoHub, and FooDB, and a novel inventory of BFIs in blood, urine, or other tissues/biofluids called Food Biomarker Alliance (FoodBAll) have been developed. Since, precision nutrition cannot deliver on its promises unless there is an accurate dietary assessment, databases containing many foods and their metabolites in humans should be developed in different populations to expand the use of metabolomics to identify BFIs in dietary assessment [114•].

Precision nutrition approaches require a thorough comprehension of how genetic-metabotype-diet interactions influence dietary biomarker levels. Genetic variations, such as SNPs, can significantly impact metabolic differences and influence dietary requirements and responses to various diets. Considering these interactions, BFIs may potentially play an important role in precision nutrition interventions by providing objective and accurate measurement of dietary habits and nutrient intake. With the identification of specific BFIs, individual responses to dietary interventions can be predicted, thereby enhancing the effectiveness of precision nutrition interventions, and leading to improved health outcomes [118]. Nonetheless, the limited utilization of BFIs into precision nutrition studies indicates a gap that requires further exploration and integration into future research endeavors, emphasizing the challenges associated with their incorporation [119•]. While there is ongoing debate regarding whether omics technology is superior to traditional biochemical markers in guiding precision nutritional assessment, the most critical challenge lies in determining which genetic variants or potential BFIs to prioritize [120]. Although BFIs provide more objective results than traditional nutritional assessment methods, the metabolomics approach has some limitations such as being more expensive and invasive, not representing the usual long-term intake, may not be sensitive or specific to the relevant food or nutrient intakes, may not be reproducible, variations may exist between ethnic groups and the most importantly, there are still no valid biomarkers for every food and nutrients [87, 121]. Therefore, future metabolomics research should concentrate on discovering novel biomarkers of specific food intake and validating these candidate biomarkers against eight validity criteria: dose-response, time-response, plausibility, reproducibility, reliability, robustness, analytical performance, and stability, and overcoming existing barriers to their application in precision nutrition studies [122].

## Microbiome-based Analysis

The ratio of human to bacterial cells within the human body is approximately 1:1, these microbial members can encode 150 times more genes than the human genome. Microbial DNA can be passed from parent to child, thus making the microbiome our "second genome" [123]. Diet is a significant modifiable factor that influences the composition of the human gut microbiota, and its modulation with prebiotics, probiotics, and postbiotics, are well-known strategies for improving health. Since, obesity results from persistent positive energy balance due to increased energy intake and/or decreased energy expenditure, traditional weight-loss therapies have relied on both hypocaloric diets and increased physical activity. Yet, the genetic and/or epigenetic structure of each individual and the uniqueness of energy metabolism controlled by mechanisms related to the microbiota are mostly ignored. Findings suggest that gut microbiota profile might contribute to explaining some of these interindividual differences to the same diet for weight loss [124]. Adults with overweight or obesity and prediabetes participating in the large multicenter clinical research, baseline gut microbiota characteristics could explain roughly 25% of the change in total body fat, after following 8 weeks of a low-energy diet providing 800 kcal to 1200 kcal per day for weight loss [125•].

Gut dysbiosis, which particularly causes short-chain fatty acid (SCFA)-producing bacteria depletion, is associated with the etiology of various diseases by inducing inflammation and disrupting gut integrity and function [126•]. In addition, Canfora et al. found that rectal SCFA mixtures including acetate, propionate, and butyrate administration decreased lipolysis and increased fatty acid oxidation, energy expenditure, and plasma peptide YY concentration in normoglycemic participants with overweight and obesity [127]. O'Grady and Shanan, highlighted that more attention needs to be paid to the details of food-microbe interactions for precision nutrition to deliver on its promise [128].

The week-long continuous monitoring of glucose levels in an 800-person cohort, along with measurement of responses to nearly 47,000 meals, revealed significant variability in response to identical meals, suggesting that the effectiveness of general dietary recommendations may be limited. Furthermore, a machine-learning algorithm was developed that accurately predicted the postprandial glycemic responses, integrating blood parameters, dietary habits, anthropometrics, physical activity, and gut microbiota from this cohort [129••]. Similarly, great interindividual variability was observed in the postprandial responses of blood glucose, triglycerides, and insulin following the same meals in the Personalised REsponses to DIetary Composition Trial (PREDICT) 1, which included over 1,000 healthy adults in the UK. Importantly, individual factors, such as the deep metagenomic sequencing of gut microbiomes, have been shown to have a greater impact on metabolic responses than meal macronutrients for postprandial lipemia, while for glucose, meal macronutrient composition explained the greatest proportion of variance in response. Using multi-omics data with the PREDICT 1, a machine-learning model was developed that predicts both postprandial glycemic and triglyceride responses. Also, Berry et al. reported that the gut microbiome deep metagenomic sequencing can be used to categorize generalizable health levels in individuals even without clinically evident disease [130].

The Diet Intervention Examining the Factors Interacting with Treatment Success (DIETFITS) trial, which included 609 adults with overweight or obesity in the U.S. and randomly assigned participants to either a low-carbohydrate or low-fat diet for 12 months, showed that the weight change between groups did not differ significantly. No associations were found between genotype patterns or baseline insulin secretion and the dietary effects on weight loss. Moreover, among the 263 proteins examined at baseline, only FGF-21 (Fibroblast growth factor-21) was identified as a predictor of weight loss, with none contributing to personalized dietary recommendations [131, 132]. While the PREDICT 1 and DIETFITS investigate the relationship between dietary response and genetic differences, the NOW (Nutrigenomics, Overweight/Obesity and Weight Management), POUNDS LOST (Prevention of Obesity Using Novel Dietary Strategies), and Food4Me (Proof of Principle) trials show the relationship between diet adherence and genetic differences [132–135].

Chen et al. reported that diet and gut microbiome play a more dominant role than genetics in explaining interindividual variability in metabolism by assessing 1,183 plasma metabolites. They emphasized that there is a significant correlation between diet quality predicted by using a machine learning-based prediction model through an individual's plasma metabolome, and the diet quality predicted by the FFQ [136•]. With omics technology, the gut microbiome can be used as a predictive indicator of interindividual variability in response to the same dietary pattern. As a result, identifying the microbiomics involved in altering human gut homeostasis, with a new sequencing approach revealing possible microbial genes, might open the way to the development of tailored treatments [137].

On the contrary, inaccurate results can be achieved if the wrong variants are selected [120, 132]. The likelihood of getting false positives is enhanced by considerable interindividual variation in microbiota composition and population-wide variances in human lifestyle [138]. Although these clinical trials have offered some clues for future studies, available data in the microbiomics field remain limited. Regarding the type of microbiome data employed, most studies have relied on 16S rRNA gene sequencing data for microbial profiling. Despite the growing accessibility of metagenomic data from whole-genome sequencing, its application in precision nutrition trials is not yet widespread [137, 139]. Biesiekierski et al. suggested that there is still insufficient evidence to support baseline microbiota as a reliable predictor of body weight and weight loss [140]. A major challenge is a limited concordance between research studying the role of the microbiota in obesity pathophysiology, limiting the ability to determine causal links between microorganisms and body weight. Since finding or selecting the right variants is subtle, variant calling algorithms with large-sample cohorts are necessary to match obesity gene mapping.

## Tailored Interventions for Weight Loss

By objective and real-time (or continuous) monitoring of individuals’ dietary intake, physical activity, physiological parameters (heart rate, blood glucose levels, sleep), or gut microbiota composition, precision nutrition has immense potential to improve the effectiveness of obesity management [141]. Previous studies assessing personalized interventions or monitoring through wearable sensors in motivating people to change their diet or lifestyle behaviors have been inconsistent. The result of the Food4Me study, which included 1,279 adults across seven European countries, indicates that personalized nutrition advice increased diet quality. Although receiving personalized nutrition advice resulted in a decrease in red meat, saturated fat, and salt consumption and an increase in folate intake, knowledge of *MTHFR* genotype did not significantly improve dietary folate intakes [133, 142, 143]. Hietaranta-Luoma et al. reported that genotype-based recommendations resulted in greater improvement in dietary fat quality in the *ApoE* Ɛ4+ group than in the *ApoE* Ɛ4- group or controls [144].

de Hoogh et al. reported that dietary intake of energy, carbohydrates, sugar, total fat, saturated fat, and polyunsaturated fatty acids decreased after personalized nutrition intervention. Additionally, participants’ BMI, body fat, and hip circumference; total cholesterol and low-density lipoprotein(LDL)-cholesterol levels decreased [145]. A meta-analysis of randomized controlled trials found that consumer wearable activity trackers-based interventions significantly increased physical activity while significantly decreasing waist circumference, LDL-cholesterol level, and systolic blood pressure in individuals with chronic diseases [146]. By contrast, a meta-analysis of randomized controlled trials, including vignette studies, revealed that genetic risk communication had no appreciable impact on the motivation to lose weight or on dietary or physical activity changes [147]. Similarly, another meta-analysis evaluating the effect of genotype-based dietary or physical activity advice in changing behavior also showed no significant difference from traditional advice [148]. Moreover, among European adults from Food4Me study, no significant physical activity changes were found during the 6-month intervention after disclosing *FTO* risk [149].

A meta-analysis evaluating nutrigenetic studies has shown that the Mediterranean diet can have a protective role against obesity, type 2 diabetes, and metabolic syndrome [150]. The long-term adherence to the Mediterranean diet may potentially lead to the mitigation of specific genes' obesogenic effects, although this assertion remains speculative. Analytical methods in nutrigenetics and nutrigenomics have used genetic risk score algorithms to capture the effects of genes and nutrients or dietary patterns on disease susceptibility. Examples of this modeling approach have been shown to have a higher risk of developing obesity among individuals at high genetic risk scores (having ≥ 7 SNPs) following a Western diet. In addition, it has been shown that dietary fiber and vegetable protein intake have protective effects, especially in low-risk individuals (< 7 SNPs) [151]. Thanks to recent advances in genomics, metabolomics, and the gut microbiome, as well as wearable technologies, precision nutrition holds out solutions to obesity [152]. Through converting genomics data into molecular phenotypes with multi-omics technology, long-term adjustments can be achieved. Further along the road, it might even be possible to establish recommended daily intake or safe upper limits for subgroups based on the individual’s genomic risk profile, as well as design appropriate screening and follow-up tools, to assess and monitor nutritional status and interindividual response to dietary intervention [6, 39].

The randomized controlled trials on weight loss with precision nutrition approaches in overweight and obese individuals are provided in Table 3. Among the five identified studies, each trial employed varying levels of personalization, including dietary recommendations based on body weight, phenotype, and genotype. In total, analyses of 5, 35, 95, and 10 SNPs were conducted as part of the genotypic information aspect by Celis-Morales et al. [153], Aldubayan et al. [155••], Cuevas-Sierra et al. [156•], and Höchsmann et al. [157], respectively. Some of these obesity-associated SNPs (*IL-6* rs1800795, *MTHFR* rs1801133, *PPARG* rs1801282, *FABP2* rs1799883, *ADRB3* rs4994, *UCP2* rs659366, *FTO* rs9939609, and *MC4R* rs17782313) had previously been presented in Table 1. Notably, the study by Celis-Morales et al. demonstrated a significant reduction in body weight among *FTO* risk carriers in the genetic group compared to the control group, emphasizing the potential efficacy of genotype-based interventions [153]. However, other trials did not report significant differences in weight loss percentage or other anthropometric measurements across different levels of personalization [154•, 155••, 156•, 157]. These diverse findings underscore the complexity of precision nutrition interventions for weight loss and highlight the need for further randomized controlled trials to elucidate optimal strategies for tailored dietary recommendations.

**Table 3:** Precision nutrition approaches for weight loss from randomized controlled trials

**Participants**	**Country**	**Year**	**Intervention**	**Duration**	**Outcome**	**Reference**
Age: 43.3 years Women: 51% BMI: 25–61 kg/m^2^ (29.3 kg/m^2^)	Germany, Greece, Ireland, the Netherlands, Poland, Spain, and the United Kingdom	2017	(1) Level 0: Control group (n = 171) Standard dietary and physical activity advice (2) Level 1: Dietary group (n = 153) Personalized dietary and physical activity advice based on body weight (3) Level 2: Phenotype group (n = 173) Personalized dietary and physical activity advice based on body weight and phenotype (waist circumference, blood cholesterol) (4) Level 3: Genetic group (n = 186) Personalized dietary and physical activity advice based on body weight, phenotype (waist circumference, blood cholesterol), and genotype (5 SNPs)	6 months	Significant decrease in body weight in *FTO* risk carriers at level 3 compared to control group (P = 0.045) No significant differences in changes in weight loss percentage, and waist circumference between level 1, 2 and 3 (P > 0.05)	Celis-Morales et al. [153]
Age: 58 ± 11 years Women: 66.8% Race: 54.3% White BMI: 27–50 kg/m^2^ (33.9 kg/m^2^) Comorbidity: prediabetes or controlled T2DM	United States of America	2022	(1) Personalized diet based on estimated postprandial glucose response using a machine learning algorithm, with utilizing a mobile app for color-coded meal scores (n = 105) (2) Standardized low-fat diet (< %25 of EI) Self-monitoring for diet, and physical activity using mobile app (n = 99)	6 months	No significant differences in changes in weight loss percentage, fat mass, fat-free mass (P = 0.16; P = 0.21; P = 0.07, respectively) Personalized diet group demonstrated higher adherence to self-monitoring (P = 0.01)	Popp et al. [154•]
Age: 45 ± 12 years Women: 69% BMI: 27–40 kg/m^2^ (32 kg/m^2^)	Denmark	2022	(1) Personalized diet + personalized behavioral change (n = 49) (a) Carbohydrate cluster (b) Lipid cluster (c) Inflammation cluster (d) Oxidative stress cluster (e) Microbiota cluster The clusters they are assigned to indicate disrupted metabolism based on participants’ metabolome analysis of 51 biomarkers such as TMAO, and their collective genotypes across 35 SNPs, with the carbohydrate cluster, for example, suggesting impaired glucose metabolism, enriched by personalized dietary intervention emphasizing high-fiber intake (2) Control diet + generic neutral recommendations (n = 51)	10 weeks	No significant differences in changes in body weight, fat mass, fat-free mass, waist circumference (P = 0.77; P = 0.77; P = 0.39; P = 0.67, respectively) No significant improvement in health parameters such as blood pressure, lipid profile, glucose metabolism, inflammatory markers (P > 0.05)	Aldubayan et al. [155••]
Age: 18–67 years Women: 70% BMI: 25–40 kg/m^2^	Spain	2022	Personalized diet based on microbiota and genetic scores (95 SNPs) (1) Moderately high-protein (MHP) diet (40% carbohydrates, 30% protein, 30% fat) (2) Low-fat (LF) diet (60% carbohydrate, 18% protein, 22% fat)	4 months	Significant differences in changes in body weight, waist circumference, visceral fat mass, LDL-cholesterol, triglycerides between men and women assigned to LF (P = 0.001, P = 0.04, P < 0.001, P = 0.003, and P = 0.008 respectively) Significant differences in changes in hip circumference and visceral fat mass between men and women assigned to MHP diet (P = 0.003, and P < 0.001, respectively) No significant differences in changes in body weight, waist circumference, hip circumference, fat mass and visceral fat mass between MHP and LF diet (P > 0.05)	Cuevas-Sierra et al. [156•]
Age: 54 ± 13 years Women: 84% Race: 68% White BMI: 27–47.5 kg/m^2^ (34.9 kg/m^2^)	United States of America	2023	Participants classified as fat-responders or carbohydrate-responders based on their collective genotypes across 10 SNPs (1) Fat-responders receiving high-fat diet (n = 44) (2) Fat-responders receiving high-carbohydrate diet (n = 41) (3) Carbohydrate-responders receiving high-fat diet (n = 21) (4) Carbohydrate-responders receiving high-carbohydrate diet (n = 16) *High-fat diet (40% fat, 45% carbohydrates; rich in unsaturated fat) *High-carbohydrate diet (65% carbohydrates, 25% fat; rich in whole-grains)	12 weeks	No significant differences in changes in weight loss percentage, body fat, waist circumference, and hip circumference between the genotype-concordant and genotype-discordant diets (P = 0.60)	Höchsmann et al. [157]

*BFI* Biomarkers of food intake, *BMI* Body mass index, *EI* Energy intake, *LDL* Low density lipoprotein, *LF* Low-fat, *MHP* Moderately high-protein, *NR* Not reported, *SNPs* Single nucleotide polymorphisms, *T2DM* Type 2 diabetes, *TMAO* Trimethylamine N-oxide

## Challenges for Clinical Use and Future Prospects

While precision medicine can currently be applied for predictive genomics in specific monogenic disorders such as cystic fibrosis, the integration of precision nutrition into healthcare systems is still in its early stages and has not yet been implemented [158]. It raises economic, legal, ethical, and social issues, including the participation of children, and the collection and storage of biological samples [159]. In a study aiming at assessing the perceptions of nutritional genomics by hospital-based registered dietitians or nutritionists, cost and ethics were identified as two major aspects that must be addressed before integrating gene-based recommendations into clinical practice [160•]. We believe that registered dietitians or nutritionists should be equipped to provide nutritional advice based on genetics with continuous learning.

The prevalence of analyzed SNPs varies considerably among different ethnic backgrounds, and the relationship with the identified phenotype cannot always be confirmed in populations with different ethnicities, limiting the application of nutrigenetics [19]. To strengthen the scientific aspect of precision nutrition, risk mapping, reliable polymorphism banks, clinically applicable epigenetic assessment, and the development of new technologies (bioinformatics) for the identification and analysis of valid, specific, and sensitive biomarkers including BFIs are required [6•]. Also, companies must meet certain standards before genetic testing can be commercialized. Data from large population-based epidemiological studies are needed to establish reliable gene banks for different genders, age groups, and ethnicities, and to better understand the obesity-gene relationship [158]. A notable example of this is the UK Biobank, a prospective cohort study involving more than 450,000 participants [161].

## Conclusion

Although different nutritional recommendations and dietary guidelines were developed for different nations, different age groups (infants, older adults), or special groups (athletes, vegetarians, or those with chronic diseases), these recommendations do not consider the wide interindividual differences. While precision nutrition offers a highly personalized perspective, the number of studies conducted so far is quite limited, and there are studies indicating no superiority over traditional dietary recommendations. Despite recent technological advancements enabling the detection of obesity-related genetic polymorphisms and the prediction of postprandial metabolic responses, the integration of precision nutrition into routine clinical practice requires further validation and refinement. It appears imperative to consider exploring the integration of various 'omics' technologies, such as genomics, metabolomics, and microbiomics, along with objective dietary assessment data, for the potential enhancement of future predictive models of individual responses to diet. Moreover, precision nutrition practices might exacerbate health disparities, as only a small percentage of the population will have access to them. Therefore, as healthcare professionals, we must not lose sight of improving the overall public health and not ignore the impact of population-based effective policies and regulations.
